# Editorial: Transition care in primary immunodeficiencies

**DOI:** 10.3389/fimmu.2024.1467284

**Published:** 2024-08-19

**Authors:** Siobhan Oisin Burns, Nizar Mahlaoui

**Affiliations:** ^1^ Institute of Immunity and Transplantation, Division of Infection and Immunity, University College London, London, United Kingdom; ^2^ Royal Free London National Health System (NHS) Foundation Trust, London, United Kingdom; ^3^ Pediatric Immuno-Hematology and Rheumatology Unit, Necker-Enfants University Hospital, Assistance Publique-Hôpitaux de Paris (AP-HP), Paris, France; ^4^ French National Reference Center for Primary Immunodeficiencies (CEREDIH), Necker-Enfants University Hospital, Assistance Publique-Hôpitaux de Paris (AP-HP), Paris, France; ^5^ Transition Care platform for Adolescents and Young Adults with Rare or Chronic Diseases « La Suite », Necker-Enfants University Hospital, Assistance Publique-Hôpitaux de Paris (AP-HP), Paris, France

**Keywords:** primary immunodeficiencies, transitional care, inborn errors of immunity (IEI), continuity of care, adolescents and young adult (AYA)

Transition has been defined as ‘a purposeful, planned process that addresses the medical, psychological, and educational/vocational needs of adolescents and young adults (AYA) with chronic physical and medical conditions as they move from child-centered to adult-oriented health care systems’ (1).

Inborn Errors of Immunity (IEI) are chronic rare disorders that result in impaired, excessive or complex dysregulated immune responses. Primary Immunodeficiencies (PID) represent the majority of IEI and are frequently diagnosed during childhood or adolescence. It has now been well established that, despite tremendous improvements in patient diagnosis, treatment and survival, complications occur throughout the life course of patients with all forms of PIDs (2), requiring long term multidisciplinary management. While transitioning from pediatric care setting to adult services, patients, caregivers, but also healthcare professionals may face, sometimes unforeseen, challenges as clinical phenotype is highly variable and not always predictable and natural history data is sparse in this group of rare diseases.

Data on transition (especially evidence-based) and recommendations in the field of PID/IEI are scarce. A recent survey published by the ERN RITA in 2023 (3) demonstrated that transition services for children with IEI in Europe are available in many countries. Approximately 75% of PID services in Europe reported having a defined transition process. In this Research Topic, Chan et al. from Southeast Asia contrasted transition care in the context of different healthcare systems. Almost half the countries in their study reported having no practice of transition care. One of the major challenges was the reluctancy of patients and caregivers to adapt to unfamiliarized adult healthcare systems. Inadequate ratio of adult immunologists and facilities for transfer as well as lack of training in transition care were also barriers to smooth transition.

It is now well established that an effective process is of utmost importance to improve long term outcome of AYA with chronic conditions. Timing and progression of transition preparation during adolescence is key. Some tools assessing youth readiness for transition are available (mostly in English language). However, both the study by Chan et al. and the previous ERN RITA survey highlighted lack of formal transition guidelines for PID as a gap that needs to be addressed (3). In this Research Topic, Mejía González et al., propose a framework for transition of patients with combined immunodeficiencies with syndromic features, who typically have complex chronic conditions adding to the challenges of ensuring a smooth transition pathway. Adult services often have less experience with this group of conditions which require extensive multidisciplinary care across hospital and community settings. The authors recommend a PID-Transition Process Coordinator and a PID-Transition Case Manager as cornerstones of the transition process.

Building on considerations for specific groups of PID patients, Alderson et al., provide insight for transitioning young adults with learning disabilities. The authors emphasize the importance of supportive interactions from clinicians and family members so that those with both a learning disability and an immunodeficiency can have a transition process that accommodates their specific requirements. The need for medical professional training as well as ongoing psychological support for adolescents with learning disabilities and their families are key for improving the transition experience in this group of PID.

To date, there is relatively little data about patient experience of transition in PID. In this Research Topic, two studies address this gap. Ouimet et al. from Montréal, Quebec, Canada examine the common transition difficulties for IEI patients with chronic illness which encompassed challenges of changing health care teams as well as issues around developing independence. The authors outline a set of recommendations reported by youths and their families. These include having a joint clinic with both pediatric and adult team, meeting health care professionals from the adult clinical unit, providing information about the adult healthcare setting (including having clear instructions on how and who to contact in case of emergencies). A transition coordinator (such as a dedicated nurse) and connection with a network of youth (and caregivers) who transitioned recently for peer meetings were also recommended. Self-advocacy and medication management were reported by King et al. as areas of challenge identified by patients during transition. Their study additionally highlighted the specific aspect of unnecessary and costly duplication of investigations (especially laboratory testing) in new transitioning patients. They suggest that implementing a formal transition protocol could minimize these costs (8). These studies highlight that further research to understand the parents’ and caregivers’ point of view, in order to inform improvements in transition for PID and to develop formal guidelines, is needed.

Contributions of multidisciplinary health care professionals — pediatric and adult teams, clinical psychologists, and ethicists — alongside contributions of colleagues from different health care systems greatly enriched the perspectives on this topic, highlighting the importance of standardized integration of transition practice towards betterment of transiting PID patients into adulthood.
